# Compressive strength and the effect of duration after photo-activation among dual-cure bulk fill composite core materials

**DOI:** 10.12669/pjms.325.10727

**Published:** 2016

**Authors:** Fahad Alkhudhairy, Fahim Vohra

**Affiliations:** 1 Fahad Alkhudhairy, Assistant Professor, Department of Restorative Dental Sciences, College of Dentistry, King Saud University, Riyadh 11545, Saudi Arabia; 2 Fahim Vohra, Associate Professor, Prosthetic Dental Science Department, College of Dentistry, King Saud University, Riyadh 11545, Saudi Arabia

**Keywords:** Bulk fill, Compressive strength, Dual cure, Zirconia nano particle, Photoactivation duration

## Abstract

**Objectives:**

To assess compressive strength and effect of duration after photoactivation on the compressive strength of different dual cure bulk fill composites.

**Methods:**

Seventy-two disc shaped (4x10mm) specimens were prepared from three dual cure bulk fill materials, ZirconCore (ZC) (n=24), MulticCore Flow (MC) (n=24) and Luxacore Dual (LC) (n=24). Half of the specimens in each material were tested for failure loads after one hour [MC1 (n=12), LC1 (n=12) & ZC1 (n=12)] and the other half in 7 days [MC7 (n=12), LC7 (n=12), ZC7 (n=12)] from photo-polymerization using the universal testing machine at a cross-head speed of 0.5 cm/minutes. Compressive strength was calculated using the formula UCS=4f/πd^2^. Compressive strengths among different groups were compared using analysis of variance (ANOVA) and Tukey’s multiple comparisons test.

**Results:**

Maximum and minimum compressive strengths were observed in ZC7 (344.14±19.22) and LC1 (202.80±15.52) groups. Specimens in LC1 [202.80 (15.52)] showed significantly lower compressive strength as compared to MC1 [287.06 (15.03)] (p<0.01) and ZC1 [276.82 (11.51)] (p<0.01). ZC7 [344.14 (19.22)] specimens showed significantly higher (p<0.01) compressive strengths compared to LC7 [324.56 (19.47)] and MC7 [315.26 (12.36)]. Compressive strengths among all three materials were significantly higher (p<0.01) at 7 days as compared to one hour.

**Conclusions:**

Bulk fill material with Zr nano-hybrid filler (ZC) showed high compressive strength compared to MC and LC. Increasing the post photo-activation duration (from one hour to 7 days) significantly improves the compressive strengths of dual cure bulk fill material.

## INTRODUCTION

Resin composites for direct restorations are globally used due to their conservative properties along with good esthetics and ease of workability.^1^ Composite restorative materials are primarily a combination of inorganic fillers and polymeric resins. Physical and mechanical properties of composites including, compressive and flexural strength, wear resistance and hardness is mainly decided by the filler particle type, composition, size and weight percentage.^2^-^6^ Moreover, increasing the filler amount improves esthetics and imparts radiopacity, in addition to reduction of polymerization shrinkage and coefficient of thermal expansion.^7^-^9^ Multiple filler combinations have been employed in resin composites with varying compositions (silica, quartz and glass), configurations (micro and macro particles) and resin-filler ratio. Introduction of nano-particles (25nm to 75nm) as fillers has allowed improvements in filler loads reaching nearly 80% in contemporary composites.^10^ Due to small filler particle size, composites with nano-particles and aggregates give improved compressive and flexural strengths, and are being used for posterior restorative composites.^3^,^11^,^12^

Zirconia (Zr) is an exceptionally stable and durable material, with excellent biocompatibility and mechanical properties.^13^-^15^ Nano-fillers are frequently made from zirconia (Zr) particles, which have particle size and crystal size of 1.5-2μm and 6nm respectively. Although Zr filler containing restorative materials have been introduced to restorative dentistry from some time,^16^ the use of Zr fillers in contemporary bulk fill dual-cure core build-up materials is novel. Bulk fill materials with more than 5% of Zr filler (ZirconCore, Harvard Dental International, GmbH, Hoppegarten, Germany) are introduced to allow for ease of core restoration placement in a single increment (low viscosity), adequate restorative adaptation and improved mechanical properties (strength from Zr particles and fraction %).

One of the leading causes of composite failures is clinical fracture. Fractures are related to the mechanical properties of composite core materials including compressive and flexural strengths. A multitude of factors such as, quality and depth of cure, light source, properties of light curing source, filler matrix ratio, filler quantity and particle size have an impact on the compressive strength of these materials.^17^ In addition, dual cured resin core build up materials have shown improved depth of cure, monomer to polymer conversion and polymerization, due to the effect of continued chemical curing after photoactivation.^17^ Therefore it is hypothesized that the incorporation of high Zr nano-particle filler content in bulk fill composite build-up material and the increase in post photoactivation duration of dual cure bulk fill resin composite azsmay improve compressive strength and other mechanical properties hence of these materials. Moreover, to our knowledge from indexed literature, evidence with regards to the effect of continued chemical cure process (duration after photoactivation of resin) on dual cure bulk fill build-up materials is limited.

Therefore the aim of the study was to assess compressive strength and the effect of duration after photoactivation on the compressive strength of a novel dual cure Zr nano-particle containing bulk fill resin composite.

## METHODS

In the present study compressive strength of a novel dual cure Zr containing nano-hybrid (bulk fill) resin composite (ZirconCore, Harvard Dental International, GmbH, Hoppegarten, Germany), was compared to two Zr free bulk fill resin composites (MulticCore Flow, Ivoclar Vivadent Schaan Liechtenstein and Luxacore Dual, DMG America, Englewood, New Jersey). In addition, the effect of post photo activation duration (one hour and seven days) on the compressive strengths of resin based, bulk fill materials were investigated.

Seventy-two disc shaped (4x10mm) specimens were prepared from three different dual cure bulk fill resin composite materials including ZirconCore (ZC) (n=24) (Harvard Dental International, GmbH, Hoppegarten, Germany), MulticCore Flow (n=24) (MC) (Ivoclar Vivadent Schaan Liechtenstein) and Luxacore Dual (LC) (n=24) (DMG America, Englewood, New Jersey). Half (n=12) of specimens in each material (MC, ZC and LC) were tested for compressive strength after one hour and seven days after fabrication. Each specimen was fabricated using a two-part stainless steel mould, having 4mm x 10mm dimension. Glass slides were placed on top and bottom of the metal mould, and the materials was light cured for 40 sec from top and 40 sec from bottom with an intensity of 650 mWcm-^2^ (Bluephase ® C8, Ivoclar Vivadent, Schaan, Liechenstein). The study groups were classified as follows,

**Group 1- MC-1**: Multicore at one hour after photoactivation

**Group 2- LC-1:** Luxacore at one hour after photoactivation

**Group 3- ZC-1:** Zirconcore at one hour after photoactivation

**Group 4- MC-7:** Multicore at seven days after photoactivation

**Group 5- LC-7:** Luxacore at seven days after photoactivation

**Group 6- ZC-7:** Zirconcore at seven days after photoactivation

The composition of resin core build-up materials is presented in Appendix A.

All the specimens (n=72) were stored in a humidifier (one hour and 7 days) at 37°C. Half of the specimens in each material category (n=12) (MC, LC and ZC) were tested for maximum failure loads after one hour and the other half in 7 days from photo-polymerization using the universal testing machine (Model 4411; Instron Corp, Canton, Mass) at a cross head speed of 0.5 cm/min. Loads were applied in the center portion of the composite cylinders using a conical round-ended metal probe producing stresses passing through the center of the specimens. Compressive strength was calculated using the formula UCS=4f/πd^2^. Where F was the load in Newton (N), and D the diameter of the cylindrical specimen in millimeters.

The data obtained, was tabulated and assessed using SPSS (Version 18, Chicago, IL. USA) software. Compressive strengths among different groups were compared using ANOVA and multiple comparisons test. P-values less than 0.05 were considered statistically significant.

## RESULTS

Means and standard deviations of compressive strengths in the experimental groups are presented in Table-I. Maximum and minimum compressive strengths were observed in ZC-7 (344.14±19.22) and LC-1 (202.80±15.52) groups respectively (Table-I). Among the groups tested at one hour (group 1-MC-1, group 2- LC-1 and group 3- ZC-1), luxacore showed significantly lower compressive strength as compared to multicore (p<0.01) and zirconcore (p<0.01), however, multicore and zirconcore were statistically comparable (p>0.01). Among the groups tested at 7 days (group 4-MC-7, group 5- LC-7 and group 6- ZC-7), zirconcore showed significantly higher (p<0.01) compressive strengths compared to luxacore and multicore, however, compressive strengths for luxacore and multicore were statistically similar (p>0.01).

**Table-I:** Means and standard deviations of compressive strengths.

*Study Groups*	*Mean (SD)*	*Study Groups*	*Mean (SD)*
MC-1	287.06 (15.03)	MC-7	315.26 (12.36)
LC-1	202.80 (15.52)	LC-7	324.56 (19.47)
ZC-1	276.82 (11.51)	ZC-7	344.14 (19.22)

MC: multicore, LC: luxacore, ZC: zirconcore, 1: 1 hour, 7: 7 days.

Compressive strengths among all three materials (MC, LC, ZC) were significantly higher (p<0.01) at 7 days as compared to one hour (Table-II). The maximum difference in compressive strength due to duration after polymerization was observed in LC material followed by ZC and MC. For detailed comparison of means and standard deviations of compressive strength among the study groups refer to Tables I & II.

**Table-II:** Effect of material and duration on compressive strength of resin based core materials assessed in the study.

*Variable*	*Compressive strength- Mean (SD)*	*p value*
Effect of Material	MC1 vs LC1: 287.06(15.03) vs 202.80 (15.52)	<0.001 **
	ZC1 vs LC1: 276.82 (11.51) vs 202.80 (15.52)	<0.001 **
	MC1 vs ZC1: 287.06(15.03) vs 276.82 (11.51)	<0.104 ns
	MC7 vs LC7: 315.26 (12.36) vs 324.56 (19.47)	<0.673 ns
	ZC7 vs LC7: 344.14 (19.22) vs 324.56 (19.47)	<0.01 *
	ZC7 vs MC7: 344.14 (19.22) vs 315.26 (12.36)	<0.001 **
Effect of post polymerization duration	MC1 vs MC7: 287.06(15.03) vs 315.26 (12.36)	<0.001 **
	LC1 vs LC7: 202.80 (15.52) vs 324.56 (19.47)	<0.001 **
	ZC1 vs ZC7: 276.82 (11.51) vs 344.14 (19.22)	<0.001 **

MC: multicore, LC: luxacore, ZC: zirconcore, 1: 1 hour, 7: 7 days,

* Significant,

** Highly significant, ns: not significant.

## DISCUSSION

The present study was based on two hypotheses. Firstly, incorporation of Zr nano-hybrid filler content with overall higher filler (>70%) in bulk fill composites would improve its mechanical properties. Secondly, increasing the duration after photoactivation would also enhance the compressive strength of bulk fill composites. Both hypotheses were found to be true in the study, as ZC showed significantly higher compressive strength as compared to MC and LC. In addition, compressive strength for all materials significantly improved at 7 days as compared to the samples assessed after one hour.

Zr nano particle containing ZC bulk fill showed significantly higher compressive strength than MC and LC bulk fill materials. Multiple explanations can be presented in this regard. Zr has high strength and fracture toughness that is imparted in ZC bulk fill material,^18^,^19^ in which Zr filler comprises more than 5% of the filler. In addition, micro and macro size filler particles are known to leave internal flaws in the composite materials, possibly affecting the resulting compressive strength of the material.^10^,^20^ Use of nano particle size Zr fillers allows for a highly condensed filler component, whereby filling defects among macro and micro filler particles and enhancing the compressive strength.^20^ It further improves the filler/polymer chain interaction, with a favorable effect on internal stress development.^21^,^22^ In a study by Hambire and Tripathi,^12^ it was concluded that adding Zr filler to a constant volume fraction of silica and glass particle containing composite, significantly improved the compressive strength of the experimental composite material. They concluded that zirconia was a significant contributing factor in compressive strength of composite materials.

**Appendix A (Composition of materials and equipment)**

1) MultiCore Flow (Ivoclar Vivadent Schaan Liechtenstein)
  Composition: Microhybrid resin composite, the monomer matrix consists of dimethacrylate (29 wt %). The inorganic fillers are barium glass, ytterbiumtrifluoride, Ba-Al-fluorosilicate glass and highly dispersed silicon dioxide (70 wt %). Additional contents are catalysts, stabilizers and pigments (1 wt %).
2) LuxaCore Dual (DMG America, Englewood, New Jersey)
  Composition: Microhybrid resin composite, Barium glass 69%, pyrog. silica 3% in a Bis-GMA based matrix of dental resins.
3) ZirconCore (Harvard Dental International, GmbH, Hoppegarten, Germany)
  Composition: Nano hybrid composite resin, Dimethacrylates 35%, Starter 2%, Silica Filler 10%, Glass filler 55%, Zirconium dioxide 5%, Pigments 0,5%.
7) Universal testing machine (Model 4411; Instron Corp, Canton, Mass)
8) Light curing device (Bluephase ® C8, Ivoclar Vivadent, Schaan, Liechenstein)
9) Digital caliper (Stainless Steel, Series-500, Mitutoyo, USA)

This is the first study assessing post photo-activation duration on the compressive strength of dual cure bulk fill composite materials. Dual cure materials have been recommended for core build ups as they allow to bulk fill the foundation restorations (cores) with adequate working and setting time, while reducing the effect of light attenuation on the depth of cure and hence the mechanical properties of the material.^17^ It has been reported that dual cured resin materials show similar mechanical properties as well as the depth of cure for bulk fill techniques than light cure resin composites^23^,^24^ and better properties (fracture toughness, depth of cure, hardness) than chemical cure composites.^17^ Interestingly, compressive strengths among all three materials (MC, LC, ZC) in the present study, were significantly higher (p<0.01) at 7 days as compared to one hour after photo-activation. Indicating that the polymerization reaction continued for some duration after one hour of photo activation increasing the conversion of monomers to polymers. Degree of conversion of monomer to polymer has been linked to the better mechanical properties of dual cured composites^25^ and therefore could be the possible explanation for the findings in the present study.

Traditionally restorative dentists prefer to place a core restoration and perform tooth preparation for indirect restorations at the same patient visit. However, as the compressive strength of the dual cure bulk fill resin core materials is significantly less at one hour of photo-activation as compared to 7 days (as shown in the present study). It may be recommended that the tooth preparation should be delayed and performed a week after core placement in order for the restoration and hence the tooth to achieve maximum strength before treatment with a rotary abrasives i.e. tooth preparation.

It is pertinent to mention, that the outcomes of this study are limited with respect to the in-vitro testing environment, compressive strength test and the specific materials used in the investigation. However, in order to assess the clinical behavior (mechanical properties and fracture strength) of Zr nano particle containing dual cure bulk fill resins and the impact of delayed tooth preparation on teeth with foundation restorations (dual cure rein composite) further in-vivo and in-vitro studies are recommended.

## CONCLUSION

Within the limitation of the specific materials, testing methods and in-vitro environment in the study, it is concluded that, resin bulk fill material with zirconia nano-hybrid filler showed high compressive strength compared to Zr free microhybrid bulk fill materials. Increasing the post photo-activation duration (from one hour to 7 days) significantly improves the compressive strengths of dual cure bulk fill resin material.
